# Are there ecological and seasonal factors influencing the resurgence of mpox in Africa?

**DOI:** 10.4102/jphia.v15i1.823

**Published:** 2024-11-06

**Authors:** Nicaise Ndembi, Placide Mbala-Kingebeni, Banda Khalifa, Mazyanga L. Mazaba, Morẹ́nikẹ́ O. Foláyan

**Affiliations:** 1 Africa Centres for Disease Control and Prevention (Africa CDC), Addis Ababa, Ethiopia; 2 Institut National de Recherche Biomédicale, Kinshasa, Democratic Republic of Congo; 3 Johns Hopkins Bloomberg School of Public Health, Baltimore, United States of America; 4 Department of Child Dental Health, Obafemi Awolowo University, Ile-Ife, Nigeria

While mpox on the African continent has historically been predominant in Central and West Africa, we can now describe it as a significant public health challenge in Africa, characterised by its complex epidemiology and increasing case numbers over recent years. Initially identified as a zoonotic disease with limited human-to-human transmission when first detected in humans in 1970 in the Democratic Republic of Congo (DRC), mpox now exhibits evolving transmission dynamics that have raised public health concerns. Mpox can enter the body through the skin, mucosa, or respiratory droplets.^1^ Recent findings, however, indicate that the virus can be transmitted during sexual contact.^2^ In addition, more recent evidence suggests its vertical transmission from mother to child.^3^

**FIGURE 1:**
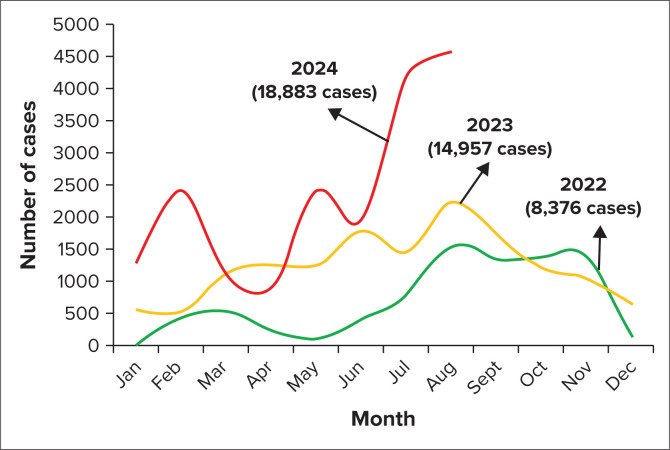
Annual trend of mpox In Africa: years 2022, 2023 and 2024.

Reports from the Africa Centres for Disease Control and Prevention (Africa CDC) indicate that from the beginning of 2022 to 18 August 2024, 42 874 mpox cases and 1512 (3.5%) deaths have been reported across 17 African countries. In 2023, cases rose to 14 957 – a 78.5% increase from the 8376 cases reported in 2022. The upward trend continued in 2024, with 18 883 cases and 541 deaths, marking a 104% increase in cases and a 10% increase in deaths compared to 2023. The reporting countries are Burundi, Cameroon, the Central African Republic, the Republic of the Congo, Côte d’Ivoire, the DRC, Gabon, Liberia, Kenya, Nigeria, Rwanda, South Africa, and Uganda. Data indicate that mpox cases tend to peak during mid-year, particularly between June and August. This consistent pattern across 3 years hints at a possible interplay between seasonal and ecological factors as influencers of the spread of mpox (Figure 1).

Broader climate change may contribute to expanding the habitats of the animal hosts and the virus. As temperatures rise and ecosystems shift, areas previously unaffected by mpox could become susceptible to new outbreaks.^4^ In Africa, seasonal variations in temperature, humidity, and rainfall can alter the habitat and behaviour of animals that serve as reservoirs for the mpox virus, such as rodents.^5^ During certain times of the year, there may be an increase in human–wildlife interactions, particularly in rural areas, which could facilitate the spillover of the mpox virus from its animal reservoirs to human populations, resulting in more zoonotic transmission.

The surge in cases observed from June to August suggests that climatic factors, such as temperature, humidity, and rainfall, may play a role in the virus’s transmission dynamics. For instance, higher temperatures and humidity levels could create more favourable conditions for the virus to survive in the environment or be transmitted by vectors. This period may also coincide with fruit seasons that attract small mammals like squirrels. For example, April to September is the citrus season in South Africa associated with the production of oranges, lemons, naartjies, grapefruits, and limes.^6^ Previous studies have suggested that prolonged climate warming often leads to the geographic spread of various infectious diseases, while weather conditions influence the timing and severity of disease outbreaks.^7,8^ These observed seasonal peaks may also coincide with periods of increased human mobility, such as during agricultural activities like hunting or farming seasons that bring humans closer to wildlife, leading to higher chances of spillover events. The interconnectedness of climate, ecological shifts, and disease spread underscores the need to address climate change as an environmental issue and a critical public health concern, emphasising the importance of understanding and providing evidence of the interconnection.

Seasonal factors might also influence healthcare-seeking behaviour and the timing of disease reporting. According to the Center for Climate and Security, people might be more prone to seeking medical care during certain times of the year, such as during colder seasons or periods of high humidity, because of a higher prevalence of illnesses or a greater awareness of health risks.^5^ For example, during certain times of the year, people might be more likely to visit health facilities, leading to increased reporting of mpox cases. Conversely, in some seasons, access to healthcare might be more limited because of logistical challenges, which could delay the detection and reporting of cases until later. Seasonal variations might affect the efficiency and intensity of disease surveillance and outbreak response efforts. During peak seasons, health systems might be more vigilant or better prepared to detect and report cases, leading to observed increases in reported cases.

Seasonal changes can also influence the viability and transmission potential of the mpox virus. For example, cooler or more humid conditions may allow the virus to survive longer in the environment, thereby increasing the likelihood of transmission. Seasonal changes in human immunity may be influenced by other circulating pathogens, nutritional factors, and gaps in public health interventions, such as lapses in preventive strategies, all of which could also play a role in how susceptible populations are to mpox infection.

The observed seasonal peaks in mpox cases in Africa are likely the result of a complex interplay of ecological factors, human behaviour, and biological factors. Currently, much attention is being paid to the biology of mpox infection. Our postulation suggests a need to analyse the annual trends in mpox cases as this can offer valuable insights into the epidemiological shifts of the disease, providing a foundation for developing targeted strategies to mitigate its impact. It can help health authorities target their efforts more effectively, such as by increasing surveillance during high-risk periods or educating the public about preventive measures when the risk of transmission is greatest.
